# Genetic Alterations Detected in Cell-Free DNA Are Associated With Enzalutamide and Abiraterone Resistance in Castration-Resistant Prostate Cancer

**DOI:** 10.1200/PO.18.00227

**Published:** 2019-04-03

**Authors:** Samantha Torquato, Aparna Pallavajjala, Alexa Goldstein, Patricia Valda Toro, John L. Silberstein, Justin Lee, Mary Nakazawa, Ian Waters, David Chu, Daniel Shinn, Taylor Groginski, Robert M. Hughes, Brian W. Simons, Hamda Khan, Zhaoyong Feng, Michael A. Carducci, Channing J. Paller, Samuel R. Denmeade, Bruce Kressel, Mario A. Eisenberger, Emmanuel S. Antonarakis, Bruce J. Trock, Ben H. Park, Paula J. Hurley

**Affiliations:** ^1^ Johns Hopkins School of Medicine, Baltimore, MD; ^2^ Johns Hopkins University, Baltimore, MD

## Abstract

**PURPOSE:**

Androgen receptor (*AR)* gene alterations, including ligand-binding domain mutations and copy number (CN) gain, have yet to be fully established as predictive markers of resistance to enzalutamide and abiraterone in men with metastatic castration-resistant prostate cancer (mCRPC). The goal of this study was to validate *AR* gene alterations detected in cell-free DNA (cfDNA) as markers of enzalutamide and abiraterone resistance in patients with mCRPC.

**METHODS:**

Patients with mCRPC (N = 62) were prospectively enrolled between 2014 and 2018. Blood was collected before therapies—enzalutamide (n = 25), abiraterone (n = 35), or enzalutamide and abiraterone (n = 2) —and at disease progression. We used deep next-generation sequencing to analyze cfDNA for sequence variants and CN status in *AR* and 45 additional cancer-associated genes. Primary end points were prostate-specific antigen response, progression-free survival (PFS), and overall survival (OS).

**RESULTS:**

Elevated tumor-specific cfDNA (circulating tumor DNA) was associated with a worse prostate-specific antigen response (hazard ratio [HR], 3.17; 95% CI, 1.11 to 9.05; *P* = .031), PFS (HR, 1.76; 95% CI, 1.03 to 3.01; *P* = .039), and OS (HR, 2.92; 95% CI, 1.40 to 6.11; *P* = .004). *AR* ligand-binding domain missense mutations (HR, 2.51; 95% CI, 1.15 to 5.72; *P* = .020) were associated with a shorter PFS in multivariable models. *AR* CN gain was associated with a shorter PFS; however, significance was lost in multivariable modeling. Genetic alterations in tumor protein p53 (HR, 2.70; 95% CI, 1.27 to 5.72; *P* = .009) and phosphoinositide 3-kinase pathway defects (HR, 2.62; 95% CI, 1.12 to 6.10; *P* = .026) were associated with a worse OS in multivariable models.

**CONCLUSION:**

These findings support the conclusion that high circulating tumor DNA burden is associated with worse outcomes to enzalutamide and abiraterone in men with mCRPC. Tumor protein p53 loss and phosphoinositide 3-kinase pathway defects were associated with worse OS in men with mCRPC. *AR* status associations with outcomes were not robust, and additional validation is needed.

## INTRODUCTION

Next-generation therapies that target the androgen–androgen receptor (AR) axis, such as abiraterone and enzalutamide, have improved survival outcomes for men with metastatic castration-resistant prostate cancer (mCRPC),^1-4^ but both primary and acquired resistance to these drugs continue to be a substantial clinical challenge. Resistance mechanisms are not fully understood; however, some forms of resistance likely involve alterations to *AR*, including amplification and ligand-binding domain (LBD) missense mutations. Although rare in primary prostate cancers,^5-7^ *AR* gene alterations are highly prevalent in mCRPC.^8-13^ Metastatic tissue biopsies as a sole means to detect and observe changes in *AR* status is impractical, and thus cell-free DNA (cfDNA) is gaining traction as a minimally invasive and easily obtainable tumor biopsy surrogate. Previous studies using cfDNA from the blood to evaluate the association of *AR* gene aberrations with resistance to abiraterone and enzalutamide are inclusive.^14-17^ *AR* copy number (CN) gain^18,19^ and/or amplification^20^ or detection of two or more *AR* mutations^20^ have been associated with worse outcomes to such therapies as abiraterone and enzalutamide. In contrast, a recent study demonstrated that neither *AR* CN gain, nor *AR* LBD mutations, were significantly associated with time to progression on abiraterone and enzalutamide therapies in multivariable models.^17^ Thus, the role of *AR* gene aberrations in mediating resistance to androgen–AR axis therapies has not been fully determined, and additional prospective studies are needed for clinical validation.

*AR* gene alterations are only detected in a subset of patients who have either primary or acquired resistance to androgen–AR therapies, thereby highlighting the need to determine other mechanisms that mediate resistance. The *AR* splice variant *AR*-V7 is associated with resistance to enzalutamide and abiraterone^21-23^ and is also associated with increased *AR* CN.^24^ In addition to *AR*, alterations in other genes, including tumor protein p53 (*TP53*), phosphatase and tensin homolog (*PTEN*), and breast cancer gene 2 (*BRCA2*), are enriched in lethal prostate cancer.^8-11^ Studies support the idea that lineage plasticity from an AR-dependent to an AR-independent state through loss of *TP53* and retinoblastoma-associated protein 1 (*RB1*) mediates resistance to AR-targeted therapies.^25-28^

Consistent with this, *TP53* defects have been shown to be associated with worse outcomes with abiraterone and enzalutamide therapies.^17^ The role of *BRCA2* and other homology-directed repair (HDR) genes in mediating resistance to enzalutamide and abiraterone has not been definitively determined. Although it has been reported that truncating mutations in *BRCA2* and ataxia-telangiectasia mutated (*ATM*) gene are associated with a shorter time to progression on enzalutamide and abiraterone,^17^ other studies have indicated that HDR defects may be associated with a better response to therapy.^29,30^

The primary goal of this study was to determine whether *AR* CN gain and/or LBD mutations detected in cfDNA were associated with enzalutamide and abiraterone resistance in patients with mCRPC. The secondary goal was to determine if alterations in other genes that are enriched in lethal prostate cancer, including *TP53, PTEN,* and *BRCA2*, were associated with response to enzalutamide and abiraterone. In this study, high circulating tumor DNA (ctDNA) burden was significantly associated with prostate-specific antigen (PSA) response, progression-free survival (PFS), and overall survival (OS). *AR* LBD mutations were associated with a shorter PFS, whereas *AR* CN gain was associated with both a shorter PFS and worse OS, but lost significance in multivariable analyses. *TP53* loss and defects in the phosphoinositide 3-kinase (PI3K) pathway were both associated with worse OS. Study limitations, including sample size and patient heterogeneity, necessitate larger and prospective validation of the association of plasma *AR* status with outcomes.

## METHODS

Patient information, study end points, sample collection, deep next-generation sequencing (NGS), sequence alignment and analysis of variants, CN variation, estimation of ctDNA fraction, and statistical analyses are found in the Data Supplement.

## RESULTS

### Patient Cohort

**FIG 1.**
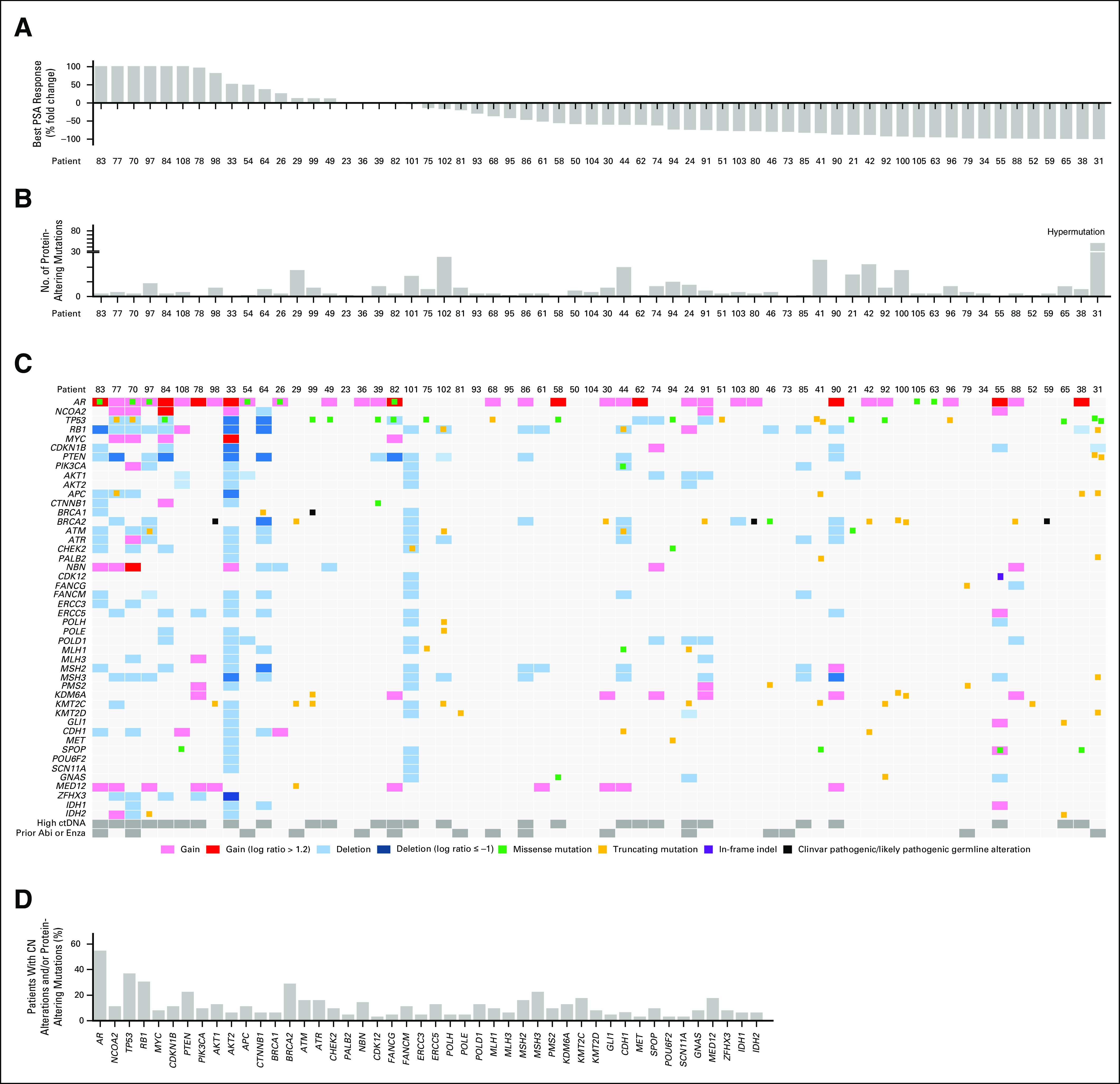
Genetic alterations detected in cell-free DNA (cfDNA) before therapy and best prostate-specific antigen (PSA) response. (A) Waterfall plot of best PSA response for all patients (N = 62) after therapy as determined by best percentage fold change in PSA. (B) Total number of protein-altering genetic changes in 46 genes detected by next-generation sequencing (NGS) of cfDNA from 62 patients before abiraterone (Abi) and enzalutamide (Enza) therapy. (C) Genetic alterations—copy number (CN) status, ClinVar pathogenic/likely pathogenic missense and germline mutations, and truncating mutations—in 46 genes detected by NGS of cfDNA from 62 patients before abiraterone and enzalutamide therapy in order of best PSA response. (D) Total number of genetic alterations—CN, ClinVar pathogenic/likely pathogenic missense and germline mutations, and truncating mutations—in 46 genes detected by NGS of cfDNA from 62 patients before abiraterone and enzalutamide therapy. AR, androgen receptor; ctDNA, circulating tumor DNA.

Patient characteristics are listed in Table 1. PSA, PSA response, and PFS were not significantly different between patients on abiraterone and enzalutamide (Table 1 and Data Supplement). Approximately one quarter of patients had received prior abiraterone or enzalutamide. Prior abiraterone or enzalutamide exposure trended toward an association for worse outcomes, including PSA response (odds ratio [OR], 2.41; 95% CI, 0.74 to 7.93; *P* = .146), PFS (hazard ratio [HR], 1.17; 95% CI, 0.63 to 2.14; *P* = .620), and OS (HR, 1.51; 95% CI, 0.71 to 3.24; *P* = .284); however, these associations did not reach statistical significance (Tables 2 and 3 and Data Supplement). ClinVar-annotated pathogenic or likely pathogenic missense mutations, truncating mutations, and/or CN alterations were detected in cfDNA from 89% of patients before therapy initiation and in 92% of patients at disease progression (Figs 1A-1D and Data Supplement).

**TABLE 1.**
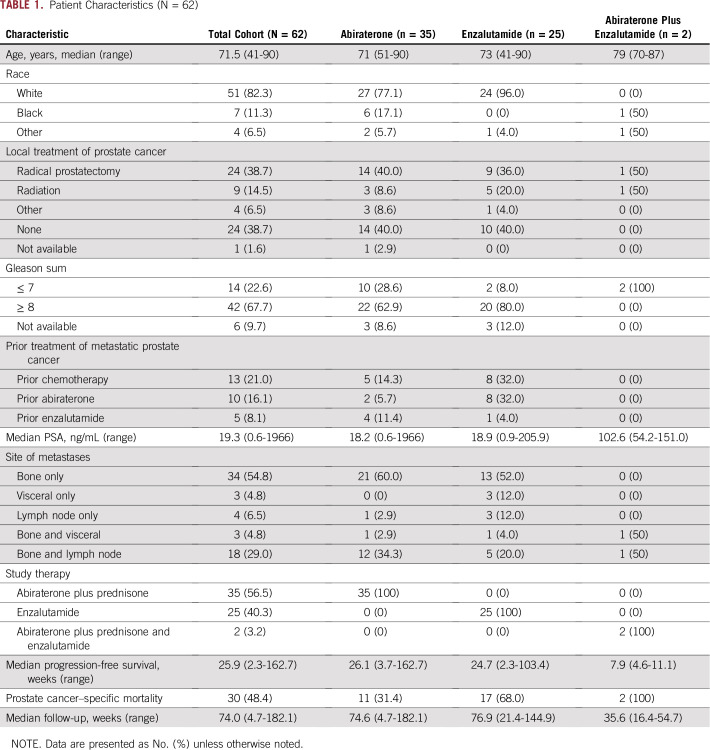
Patient Characteristics (N = 62)

**TABLE 2.**
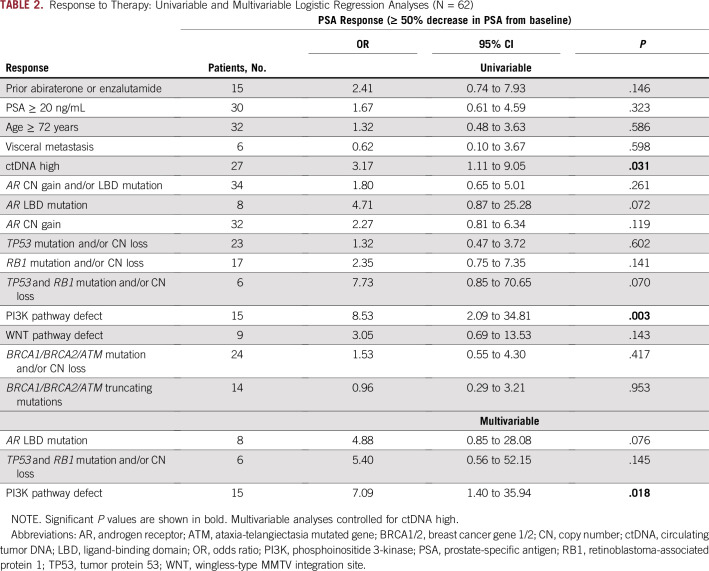
Response to Therapy: Univariable and Multivariable Logistic Regression Analyses (N = 62)

**TABLE 3.**
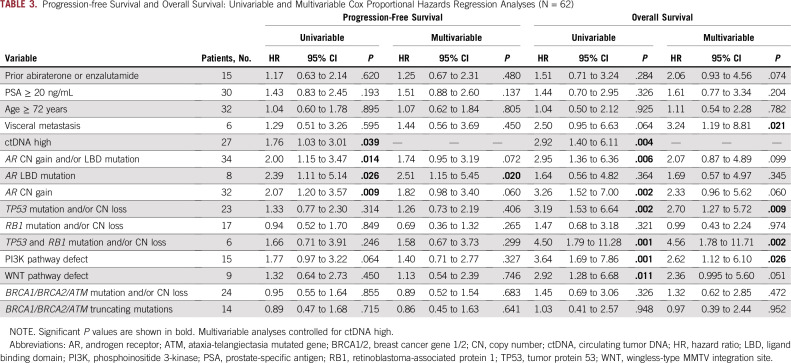
Progression-free Survival and Overall Survival: Univariable and Multivariable Cox Proportional Hazards Regression Analyses (N = 62)

### ctDNA

**FIG 2.**
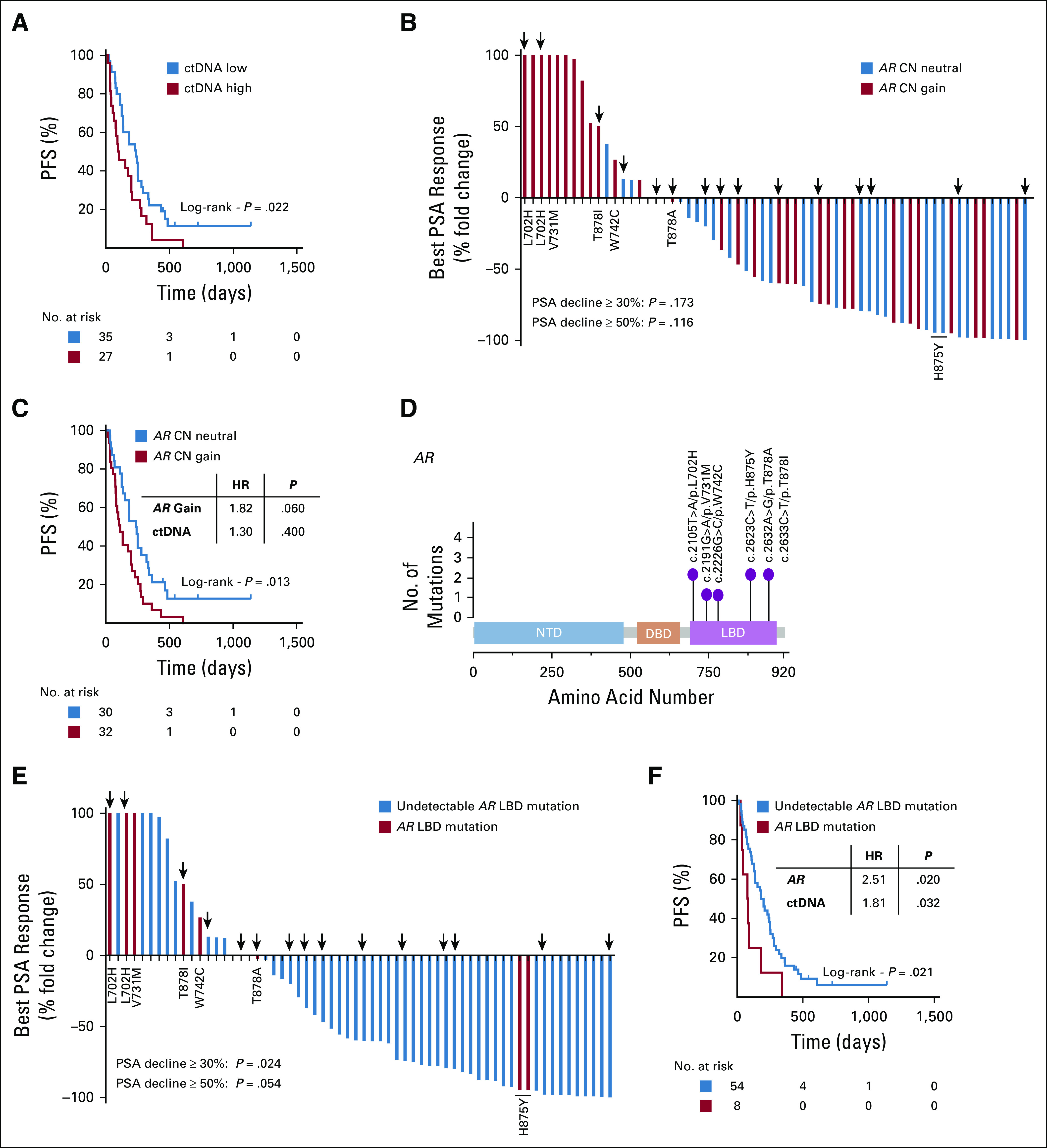
Progression-free survival (PFS): Pathogenic androgen receptor (*AR*) ligand-binding domain (LBD) mutations are associated with a shorter time to progression. (A) Kaplan-Meier method and log-rank test were used to determine median time to progression for patients who had high versus low circulating tumor DNA (ctDNA) before therapy. (B) Waterfall plot of best prostate-specific antigen (PSA) response for all patients (N = 62) after therapy as determined by best percentage fold change in PSA. *AR* copy number (CN) gain and *AR* LBD missense mutations were determined by deep next-generation sequencing (NGS) of cell-free DNA (cfDNA) before therapy. *AR* LBD missense mutations were included for patients with detectable mutations. Black arrow indicates patients with prior abiraterone or enzalutamide therapy. χ^2^ analyses for a 30% or greater and 50% or greater PSA decrease. (C) Kaplan-Meier method and log-rank test were used to determine median time to progression for patients who had a gain in *AR* CN compared with patients who were *AR* CN neutral before therapy. The association of *AR* CN gain with PFS controlled for ctDNA burden using multivariable proportional hazards regression modeling. (C) Gene schematic illustrating pathogenic *AR* LBD mutations detected by targeted NGS of cfDNA before abiraterone and enzalutamide therapies. (E) Waterfall plot of best PSA response for all patients (N = 62) after therapy as determined by best percentage fold change in PSA. *AR* LBD mutations were determined by deep NGS of cfDNA before therapy and listed below. Black arrow indicates patients with prior abiraterone or enzalutamide therapy. χ^2^ analyses for a 30% or greater and 50% or greater PSA decrease. (F) Kaplan-Meier method and log-rank test were used to determine median time to progression for patients who were positive versus negative for *AR* LBD mutations before therapy. The association of *AR* LBD mutations with PFS controlled for ctDNA burden using multivariable proportional hazards regression modeling. DBD, DNA-binding domain; NTD, N-terminal domain.

**FIG 3.**
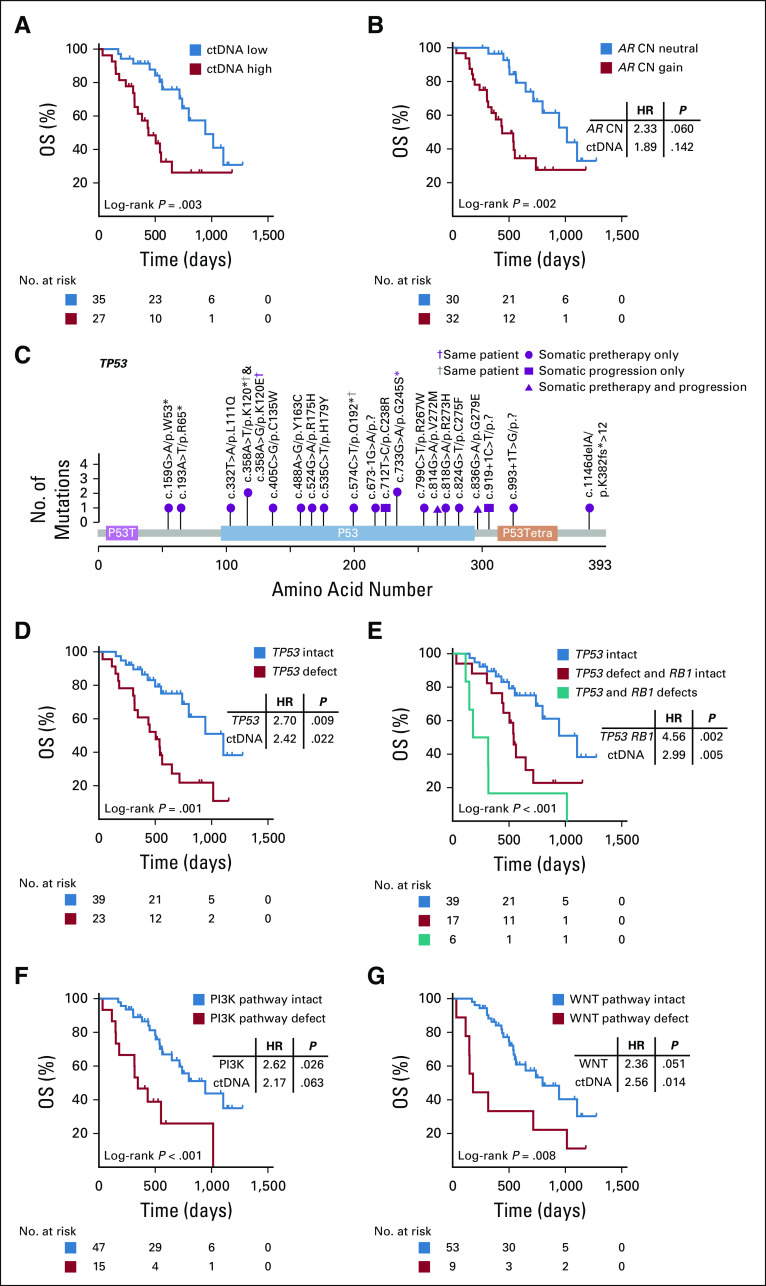
Overall survival (OS): Tumor protein p53 (*TP53*) and phosphoinositide 3-kinase (PI3K) pathway defects are associated with worse OS. (A) Kaplan-Meier method and log-rank test were used to determine median OS for patients who had high versus low circulating tumor DNA (ctDNA) before therapy. (B) Kaplan-Meier method and log-rank test were used to determine median OS for patients who had androgen receptor (AR) copy number (CN) gain before therapy. Association of *AR* CN gain with OS controlled for ctDNA burden using multivariable proportional hazards regression modeling. (C) Gene schematic illustrating deleterious *TP53* mutations detected by deep next-generation sequencing (NGS) of cell-free DNA (cfDNA) before abiraterone and enzalutamide therapies and at disease progression while on therapy. (D) Kaplan-Meier method and log-rank test were used to determine median OS for patients who had *TP53* defects—CN loss and or ClinVar pathogenic/likely pathogenic mutations—before therapy. Association of *TP53* defects with OS controlled for ctDNA burden using multivariable proportional hazards regression modeling. (E) Kaplan-Meier method and log-rank test were used to determine median OS for patients who had both *TP53* and retinoblastoma-associated protein 1 (*RB1*) defects compared with patients who had *TP53* defects but were *RB1* intact—CN loss and or ClinVar pathogenic/likely pathogenic mutations—before therapy. Association of dual *TP53* and *RB1* defects with OS controlled for ctDNA burden using multivariable proportional hazards regression modeling. (F) Kaplan-Meier method and log-rank test were used to determine median OS for patients who had PI3K pathway defects—CN loss and/or truncating mutations in phosphatase and tensin homolog and/or CN gain of *PIK3CA*—before therapy. Association of *PI3K* defects with OS controlled for ctDNA burden using multivariable proportional hazards regression modeling. (G) Kaplan-Meier method and log-rank test were used to determine median OS for patients who had wingless-type MMTV integration site (WNT) pathway defects—CN loss and/or truncating mutations in adenomatous polyposis coli and/or CN gain and/or pathogenic missense mutations in β-catenin—before therapy. Association of WNT defects with OS controlled for ctDNA burden using multivariable proportional hazards regression modeling. P53, P53 DNA-binding domain; P53T, P53 transactivation motif; P53Tetra, P53 tetramerisation motif.

Total cfDNA concentration before therapy was associated with PSA (*P* = .002; Data Supplement). We used deep NGS to analyze cfDNA for CN variation and mutations in 46 cancer-associated genes (Data Supplement). Nearly all patients (61 of 62) had detectable CN variation(s) and/or mutation(s) with an allelic frequency above the 1% cutoff before therapy (Figs 1B and 1C). High ctDNA was detected in approximately 44% of patients before therapy (Fig 1C). Consistent with previous findings,^14,17,20^ high ctDNA was significantly associated with a worse PSA response (OR, 3.17; 95% CI, 1.11 to 9.05; *P* = .031) by logistic regression analyses (Table 2). High ctDNA was associated with a significantly shorter median time to progression (14.0 weeks *v* 34.0 weeks; *P* = .022) and, using proportional hazards regression modeling, a shorter PFS (HR, 1.76; 95% CI, 1.03 to 3.01; *P* = .039; Table 3 and Fig 2A). High ctDNA was also significantly associated with a shorter median survival (62.7 weeks *v* 134.9 weeks; *P* = .003) and worse OS (HR, 2.92; 95% CI, 1.40 to 6.11; *P* = .004; Table 3 and Fig 3A). Other clinical variables, such as PSA, age, and visceral metastases, were not significantly associated with PSA response, PFS, or OS in univariable analyses (Tables 2 and 3 and Data Supplement).

### AR

Previous studies that evaluated associations between *AR* gene alterations, including CN gain and LBD missense mutations, with therapeutic outcomes are not definitive.^14,15,17-20,31^ *AR* CN gain was detected in approximately one half of patients before therapy and at disease progression (Figs 1C and 1D and Data Supplement). *AR* CN gain was not significantly associated with PSA response by logistic regression analysis (*P* = .119; Table 2 and Fig 2B), but was associated with a shorter median time to progression (16.1 weeks *v* 34.0 weeks; *P* = .013) and a shorter median survival (62.7 weeks *v* 144.9 weeks; *P* = .002; Figs 2C and 3B). Using proportional hazards regression modeling, PFS (HR, 2.07; 95% CI, 1.20 to 3.57; *P* = .009) and OS (HR, 3.26; 95% CI, 1.52 to 7.11; *P* = .002) were shorter in patients with *AR* CN gain; however, significance was lost upon inclusion of ctDNA burden in multivariable modeling (Figs 2C and 3B and Table 3).

Pathogenic *AR* LBD missense mutations were detected in cfDNA from 13% (eight of 62) of patients before therapy initiation and in an additional 15% (four of 26) of evaluable patients at disease progression (Figs 1C, 1D, and 2D and Data Supplement). Of the eight patients who had detectable *AR* LBD mutations before therapy, six did not have a PSA response, whereas two patients who harbored the H875Y mutation had PSA responses on abiraterone (Fig 2C). Using logistic regression analyses, *AR* LBD mutations were not significantly associated with a worse PSA response rate (*P* = .072; Table 2). However, pathogenic *AR* LBD missense mutations were associated with a worse 30% or more decline in PSA (OR, 6.00; 95% CI, 1.10 to 32.76; *P* = .039) that remained significant in multivariable logistic regression analyses (Data Supplement).

Median time to progression was shorter in patients who had a detectable *AR* LBD mutation than in patients without a detectable *AR* LBD mutation (11.4 weeks *v* 28.7 weeks; *P* = .021; Fig 2F). Using proportional hazards regression modeling, *AR* LBD mutations detected before therapy were associated with a shorter time to progression (HR, 2.39; 95% CI, 1.11 to 5.14; *P* = .026), even when controlled for ctDNA burden (*P* = .020) and other variables (Table 3 and Data Supplement). However, detectable *AR* LBD mutations were not significantly associated with worse OS (*P* = .364; Table 3).

*AR* CN gain and LBD mutations were not mutually exclusive in cfDNA (Fig 1C). Two *AR* mutations at different allelic frequencies—T878A at 9.4% and L702H at 1.5%—were detected in one patient who experienced disease progression on abiraterone plus prednisone who also had *AR* CN gain (Data Supplement). Studies support the idea that the *AR* L702H mutation mediates an acquired response to glucocorticoids, thereby providing rationale to switch from prednisone to dexamethasone.^14,20,32^ In support of this notion, replacement of prednisone with dexamethasone resulted in a greater than 80% PSA decline for this patient (Data Supplement).

### TP53 and RB1

Genetic alterations in *TP53* are highly enriched in lethal prostate cancer^8-11^ and have recently been shown to be associated with worse PFS and OS in patients treated with abiraterone and enzalutamide.^17^ *TP53* was highly altered in patients’ cfDNA (Figs 1C, 1D, and 3C); however, *TP53* defects—pathogenic mutations and/or CN loss—were not associated with PSA response (*P* = .602) or PFS (*P* = .314; Tables 2 and 3). Conversely, median OS was shorter in patients with a *TP53* defect compared with patients without a detectable *TP53* defect (68.1 weeks *v* 134.9 weeks; *P* = .001; Fig 3D). Using proportional hazards regression modeling, *TP53* defects were associated with worse OS (HR, 3.19; 95% CI, 1.53 to 6.64; *P* = .002) that remained significant after adjusting for clinical variables (Table 3 and Data Supplement). Patients with both *TP53* and *RB1* defects had shorter median OS compared with patients with a *TP53* defect alone or with patients with intact *TP53* (35.4 weeks *v* 77.4 weeks *v* 157.7 weeks; *P* < .001; Fig 3E). *TP53* defects in conjunction with *RB1* defects were associated with worse OS (HR, 4.50; 95% CI, 1.79 to 11.28; *P* = .001) that remained significant after adjusting for other variables (Table 3 and Data Supplement).

### PI3K and Wingless-Type MMTV Integration Site Pathways

PI3K pathway defects involving genetic alterations in *PTEN*—CN loss and/or truncating mutations—and *PIK3CA*—CN gain and/or pathogenic missense mutation—were detected in nearly one quarter of patients before therapy (Figs 1C and 1D). Patients with PI3K pathway defects before therapy had a significantly shorter median survival (49.4 weeks *v* 134.9 weeks; *P* < .001) and worse OS (HR, 3.64; 95% CI, 1.69 to 7.86; *P* = .001), even after controlling for ctDNA burden (*P* = .026; Fig 3F, Table 3, and Data Supplement). PI3K pathway alterations were also associated with a worse PSA response that remained significant after adjusting for ctDNA burden (HR, 8.53; 95% CI, 2.09 to 34.81; *P* = .003; Table 2). Wingless-type MMTV integration site pathway defects involving genetic alterations in adenomatous polyposis coli—CN loss and/or truncating mutations—and β-catenin—CN gain and pathogenic missense mutations—were detected in nearly 15% of patients before to therapy (Figs 1C and 1D). Wingless-type MMTV integration site pathway defects were associated with a worse OS (HR, 2.92; 95% CI, 1.28 to 6.68; *P* = .011) using proportional hazards regression modeling; however, significance was lost after controlling for ctDNA burden (*P* = .051; Fig 3G and Table 3).

### BRCA1, BRCA2, and ATM

Men with lethal prostate cancer are more likely to have germline mutations in DNA repair genes^33,34^; however, the association of HDR gene defects with response to abiraterone and enzalutamide is conflicting.^17,29,30^ Approximately one third of patients had germline and/or somatic deleterious mutations in or CN loss of *BRCA1*, *BRCA2*, or *ATM* before therapy, with some patients having more than one mutation (Figs 1C and 1D and Data Supplement). Collective ClinVar deleterious missense mutations, truncating mutations, and/or CN loss in *BRCA2, BRCA1*, or *ATM* were not significantly associated with PSA response (*P* = .417), PFS (*P* = .855), or OS (*P* = .326; Tables 2 and 3). Analysis of truncating mutations alone in *BRCA1*, *BRCA2*, and *ATM* did not increase prognostic significance.

## DISCUSSION

Liquid biopsies using cfDNA as a tumor analyte are rapidly being developed for cancer diagnostics of solid tumors.^35-37^ When obtained concurrently, plasma-derived cfDNA is highly concordant with tissue biopsies for tumor-specific genetic alterations.^38,39^ As a result of their advantages over traditional tissue biopsies, including the ease of accessibility for sequential monitoring of cancer dynamics and recapitulation of tumor heterogeneity, clinical development of cfDNA has the potential to advance prostate cancer precision medicine.^40^

Mechanisms of resistance to abiraterone and enzalutamide likely involve alterations to androgen–AR axis signaling. Previous studies have indicated that collective genetic aberrations to *AR,* including CN gain and mutations, are associated with worse outcomes in patients on abiraterone or enzalutamide therapies.^14,15^ The value of *AR* LBD mutations alone as a predictive marker for response to enzalutamide and abiraterone in patients with mCRPC has yet to be fully established. A previous study demonstrated that patients with mCRPC harboring two or more *AR* mutations had worse outcomes on enzalutamide.^20^ An additional retrospective study showed that *AR* mutations—L702H and T878A—were associated with shorter PFS and OS in postdocetaxel patients with mCRPC on abiraterone.^19^ In contrast, a large prospective study reported that *AR* LBD mutations were not associated with time to progression on abiraterone or enzalutamide therapies in treatment-naïve patients with mCRPC.^17^ In the current study, we found that *AR* LBD missense mutations detected in cfDNA before enzalutamide and abiraterone therapies were associated with a shorter PFS, but not PSA response or OS. Lack of a strong association with PSA response and OS lessens the likelihood that *AR* LBD mutations will be rigorous biomarkers for therapeutic decision making. Discrepancies between study findings may be a result of several factors, including prior therapies, study therapy, study design, specific *AR* LBD mutation, *AR* amplification, and disease burden. Prior therapies likely change the repertoire and incidence of *AR* LBD mutations.^19,41^ As a result of their low individual prevalence, *AR* LBD mutations are often combined for analyses; however, studies support the idea that *AR* LBD mutations have distinct functional properties, including ligand promiscuity and agonistic activity, that mediate selective-therapy resistance.^32,41^ Furthermore, the coincidence of other genetic alterations, including *AR* amplification or *TP53* defects, and overall disease burden may be confounders. Future large-scale and multicenter prospective validation will be necessary to determine fully the roles of individual mutations in drug resistance.

*AR* CN gain as a single marker has been demonstrated to be associated with worse outcomes in patients with mCRPC on abiraterone and enzalutamide.^19,20^ A retrospective study reported that *AR* CN gain was associated with worse PFS and OS in men who were treated with enzalutamide or abiraterone for mCRPC.^19^ Similarly, *AR* CN gain was also reported to be associated with a worse PSA response and PFS in patients on enzalutamide.^20^ Our study also demonstrated an association of *AR* CN gain with PFS and OS; however, significance was lost in multivariable modeling, which is consistent with a previous report.^17^ Clearly, additional prospective studies are needed to assess the clinical strength of *AR* CN gain as a predictive biomarker for therapeutic response to enzalutamide and abiraterone in patients with mCRPC.

In the current study, *TP53* and PI3K pathway defects were associated with worse OS. Deregulation of these pathways likely mediates resistance to androgen–AR axis therapies. Concurrent *TP53* and *RB1* defects are highly enriched in AR-independent neuroendocrine mCRPC compared with adenocarcinoma mCRPC.^42^ Combined *TP53* and *RB1* loss has been shown to promote lineage switching from an AR-dependent to an AR-independent state^41,43,44^ and consequent resistance to AR-targeted therapies. Similar to *TP53*, genetic alterations in *PTEN* are enriched in mCRPC compared with metastatic castration-sensitive prostate cancer and localized prostate cancer.^11^

Studies suggest that *PTEN* loss may mediate castration resistance by downregulating AR,^25-28^ thereby supporting a rationale for combined inhibition of PI3K and AR- in *PTEN*-deficient mCRPCs.^45,46^

Association of pathogenic mutations in HDR genes with response to abiraterone and enzalutamide therapy is conflicting. A clinical trial in patients with mCRPC suggested that genetic alterations in HDR genes that were detected in metastatic biopsy tissue may be associated with longer PFS when on abiraterone therapy.^29^ Concordant findings were observed in a second study that supported the idea that patients with mCRPC harboring a germline *BRCA1/2* or *ATM* mutation may also have improved outcomes to abiraterone and enzalutamide.^30^ In contrast, another study showed that truncating mutations in *BRCA2* and *ATM* detected in cfDNA were associated with a shorter time to progression on abiraterone and enzalutamide therapies in treatment-naïve patients with mCRPC.^17^ In our study, collective somatic and germline genetic alterations were also not associated with worse outcomes to enzalutamide and abiraterone. Association differences may reflect variables, such as sample size, prior treatment status, disease burden, disease heterogeneity, somatic versus germline, and single versus dual loss. Certainly, additional prospective investigation is needed to determine the clinical significance of HDR mutations as predictive markers to abiraterone and enzalutamide therapies.

In the current study, many patients had detectable alterations that could serve as potential therapeutic targets. Previous studies have shown that patients with mCRPC with either germline or somatic mutations in HDR genes achieved significant responses to olaparib^47^ and to abiraterone plus veliparib.^29^ More than one quarter of patients in our study had a deleterious germline or somatic *BRCA1*, *BRCA2*, or *ATM* mutation detected before therapy or at disease progression, which suggests that these patients may benefit from therapies that target poly (ADP-ribose) polymerase or platinum-based chemotherapy.^29,47,48^ In addition, immunotherapy trials have been largely unsuccessful in men with mCRPC^49^; however, rare responders have been reported.^50^ A seminal clinical trial demonstrated that microsatellite instable cancers caused by mismatch repair (MMR) gene deficiency were sensitive to programmed death-1 blockade, perhaps because of the formation of neoantigens resulting from increased mutational burden.^51^ Inactivation of MMR genes and elevated mutational burden have been detected in some men with aggressive prostate cancers.^33,52,53^ One patient in our study had a detectable noncanonical MMR gene mutation in his cfDNA and a correspondingly high mutational burden that suggested that he may be an ideal candidate for checkpoint immunotherapy. This study supports the idea that cfDNA may be a useful analyte for directing clinical decisions in prostate cancer precision medicine.

Several limitations to our study exist. Of note, the small sample size precluded multivariable analyses that incorporated more than two variables and analyses by therapy subgroup. Consistent with other reports, patients with prior exposure to abiraterone and enzalutamide experienced worse outcomes.^54-59^ Statistical significance was not reached, likely because of the small overall sample size and the few patients with prior therapy. In addition, the small size precluded any definitive conclusions pertaining to the association of *AR* LBD mutations with outcomes. Larger prospective studies will be needed to validate our findings. Samples were obtained from two hospitals, and future prospective studies would benefit from the inclusion of a larger number of institutions. Future prospective studies would also be strengthened by radiologic confirmation of progression for every patient. An additional limitation was the variability of cfDNA input for NGS among patients. NGS protocols were adjusted on the basis of total input, but for patients with low input the lack of genetic alteration detection was considered indeterminate as opposed to negative. In addition, mutations in such genes as *TP53* and *ATM* detected in cfDNA at low allelic frequencies may be false positives as a result of clonal hematopoiesis.^60^ Corresponding tissue was not available for all samples to confirm *TP53* status, and future studies will examine both prostate tumor tissue and blood leukocytes for genetic alterations. A final limitation was our inability to evaluate *AR* splice variants, including *AR*-V7, because of the requirement of circulating tumor cells or whole-blood RNA. The presence of AR-V7 is certainly another established mechanism of primary and acquired resistance to next-generation hormonal therapies.^21-23^ Future studies should aim to simultaneously analyze the full complement of *AR* aberrations, including gene mutations, amplifications, genomic structural rearrangements, and mRNA splice variants, from a single liquid biopsy.

In summary, our findings indicate ctDNA burden was highly associated with worse outcomes to enzalutamide and abiraterone. Association of *AR* status with outcomes was not robust and will need additional prospective validation. *TP53* loss, especially in the context of concurrent *RB1* defects, and PI3K pathway defects were associated with worse OS. These studies provide the rationale for larger prospective multi-institutional studies to additionally assess the clinical utility of integrating genetic alterations detected in cfDNA for the optimal management of metastatic prostate cancer.

## References

[1] de Bono JS Logothetis CJ Molina A et al Abiraterone and increased survival in metastatic prostate cancer N Engl J Med 364 1995 2005 2011 21612468 10.1056/NEJMoa1014618 PMC3471149

[2] Ryan CJ Smith MR de Bono JS et al Abiraterone in metastatic prostate cancer without previous chemotherapy N Engl J Med 368 138 148 2013 23228172 10.1056/NEJMoa1209096 PMC3683570

[3] Scher HI Fizazi K Saad F et al Increased survival with enzalutamide in prostate cancer after chemotherapy N Engl J Med 367 1187 1197 2012 22894553 10.1056/NEJMoa1207506

[4] Beer TM Armstrong AJ Rathkopf DE et al Enzalutamide in metastatic prostate cancer before chemotherapy N Engl J Med 371 424 433 2014 24881730 10.1056/NEJMoa1405095 PMC4418931

[5] Cancer Genome Atlas Research Network Comprehensive molecular characterization of urothelial bladder carcinoma Nature 507 315 322 2014 24476821 10.1038/nature12965 PMC3962515

[6] Baca SC Prandi D Lawrence MS et al Punctuated evolution of prostate cancer genomes Cell 153 666 677 2013 23622249 10.1016/j.cell.2013.03.021 PMC3690918

[7] Taylor BS Schultz N Hieronymus H et al Integrative genomic profiling of human prostate cancer Cancer Cell 18 11 22 2010 20579941 10.1016/j.ccr.2010.05.026 PMC3198787

[8] Grasso CS Wu YM Robinson DR et al The mutational landscape of lethal castration-resistant prostate cancer Nature 487 239 243 2012 22722839 10.1038/nature11125 PMC3396711

[9] Kumar A Coleman I Morrissey C et al Substantial interindividual and limited intraindividual genomic diversity among tumors from men with metastatic prostate cancer Nat Med 22 369 378 2016 26928463 10.1038/nm.4053 PMC5045679

[10] Robinson D, Van Allen EM, Wu YM, et al: Integrative clinical genomics of advanced prostate cancer. Cell 161:1215-1228, 2015 [Erratum: Cell 162:454, 2015] 10.1016/j.cell.2015.05.001 PMC4484602 26000489

[11] Abida W Armenia J Gopalan A et al Prospective genomic profiling of prostate cancer across disease states reveals germline and somatic alterations that may affect clinical decision making JCO Precis Oncoldoi:10.1200/PO.17.00029 10.1200/PO.17.00029 PMC5558263 28825054

[12] Cerami E Gao J Dogrusoz U et al The cBio cancer genomics portal: An open platform for exploring multidimensional cancer genomics data Cancer Discov 2 401 404 2012 22588877 10.1158/2159-8290.CD-12-0095 PMC3956037

[13] Gao J Aksoy BA Dogrusoz U et al Integrative analysis of complex cancer genomics and clinical profiles using the cBioPortal Sci Signal 6 pl1 2013 23550210 10.1126/scisignal.2004088 PMC4160307

[14] Romanel A Gasi Tandefelt D Conteduca V et al Plasma AR and abiraterone-resistant prostate cancer Sci Transl Med 7 312re10 2015 10.1126/scitranslmed.aac9511 PMC6112410 26537258

[15] Azad AA Volik SV Wyatt AW et al Androgen receptor gene aberrations in circulating cell-free DNA: Biomarkers of therapeutic resistance in castration-resistant prostate cancer Clin Cancer Res 21 2315 2324 2015 25712683 10.1158/1078-0432.CCR-14-2666

[16] Goldstein A Toro PV Lee J et al Detection fidelity of AR mutations in plasma derived cell-free DNA Oncotarget 8 15651 15662 2017 28152506 10.18632/oncotarget.14926 PMC5362513

[17] Annala M Vandekerkhove G Khalaf D et al Circulating tumor DNA genomics correlate with resistance to abiraterone and enzalutamide in prostate cancer Cancer Discov 8 444 457 2018 29367197 10.1158/2159-8290.CD-17-0937

[18] Salvi S Casadio V Conteduca V et al Circulating cell-free AR and CYP17A1 copy number variations may associate with outcome of metastatic castration-resistant prostate cancer patients treated with abiraterone Br J Cancer 112 1717 1724 2015 25897673 10.1038/bjc.2015.128 PMC4430717

[19] Conteduca V Wetterskog D Sharabiani MTA et al Androgen receptor gene status in plasma DNA associates with worse outcome on enzalutamide or abiraterone for castration-resistant prostate cancer: A multi-institution correlative biomarker study Ann Oncol 28 1508 1516 2017 28472366 10.1093/annonc/mdx155 PMC5834043

[20] Wyatt AW Azad AA Volik SV et al Genomic alterations in cell-free DNA and enzalutamide resistance in castration-resistant prostate cancer JAMA Oncol 2 1598 1606 2016 27148695 10.1001/jamaoncol.2016.0494 PMC5097690

[21] Antonarakis ES Lu C Wang H et al AR-V7 and resistance to enzalutamide and abiraterone in prostate cancer N Engl J Med 371 1028 1038 2014 25184630 10.1056/NEJMoa1315815 PMC4201502

[22] Qu F Xie W Nakabayashi M et al Association of AR-V7 and prostate-specific antigen RNA levels in blood with efficacy of abiraterone acetate and enzalutamide treatment in men with prostate cancer Clin Cancer Res 23 726 734 2017 27489290 10.1158/1078-0432.CCR-16-1070 PMC5675562

[23] Antonarakis ES Lu C Luber B et al Clinical significance of androgen receptor splice variant-7 mRNA detection in circulating tumor cells of men with metastatic castration-resistant prostate cancer treated with first- and second-line abiraterone and enzalutamide J Clin Oncol 35 2149 2156 2017 28384066 10.1200/JCO.2016.70.1961 PMC5493048

[24] Henzler C Li Y Yang R et al Truncation and constitutive activation of the androgen receptor by diverse genomic rearrangements in prostate cancer Nat Commun 7 13668 2016 27897170 10.1038/ncomms13668 PMC5141345

[25] Shen MM Abate-Shen C Pten inactivation and the emergence of androgen-independent prostate cancer Cancer Res 67 6535 6538 2007 17638861 10.1158/0008-5472.CAN-07-1271

[26] Jiao J Wang S Qiao R et al Murine cell lines derived from Pten null prostate cancer show the critical role of PTEN in hormone refractory prostate cancer development Cancer Res 67 6083 6091 2007 17616663 10.1158/0008-5472.CAN-06-4202

[27] Mulholland DJ Tran LM Li Y et al Cell autonomous role of PTEN in regulating castration-resistant prostate cancer growth Cancer Cell 19 792 804 2011 21620777 10.1016/j.ccr.2011.05.006 PMC3157296

[28] Carver BS Chapinski C Wongvipat J et al Reciprocal feedback regulation of PI3K and androgen receptor signaling in PTEN-deficient prostate cancer Cancer Cell 19 575 586 2011 21575859 10.1016/j.ccr.2011.04.008 PMC3142785

[29] Hussain M Daignault-Newton S Twardowski PW et al Targeting androgen receptor and DNA repair in metastatic castration-resistant prostate cancer: Results from NCI 9012 J Clin Oncol 36 991 999 2018 29261439 10.1200/JCO.2017.75.7310 PMC6075827

[30] Antonarakis ES Changxue L Luber B et al Germline DNA-repair gene mutations and outcomes in men with metastatic castration-resistant prostate cancer receiving first-line abiraterone and enzalutamide Eur Urol 74 218 225 2018 29439820 10.1016/j.eururo.2018.01.035 PMC6045965

[31] Salvi S Casadio V Conteduca V et al Circulating AR copy number and outcome to enzalutamide in docetaxel-treated metastatic castration-resistant prostate cancer Oncotarget 7 37839 37845 2016 27191887 10.18632/oncotarget.9341 PMC5122353

[32] Lallous N Volik SV Awrey S et al Functional analysis of androgen receptor mutations that confer anti-androgen resistance identified in circulating cell-free DNA from prostate cancer patients Genome Biol 17 10 2016 26813233 10.1186/s13059-015-0864-1 PMC4729137

[33] Pritchard CC Mateo J Walsh MF et al Inherited DNA-repair gene mutations in men with metastatic prostate cancer N Engl J Med 375 443 453 2016 27433846 10.1056/NEJMoa1603144 PMC4986616

[34] Na R Zheng SL Han M et al Germline mutations in ATM and BRCA1/2 distinguish risk for lethal and indolent prostate cancer and are associated with early age at death Eur Urol 71 740 747 2017 27989354 10.1016/j.eururo.2016.11.033 PMC5535082

[35] Cohen JD Li L Wang Y et al Detection and localization of surgically resectable cancers with a multi-analyte blood test Science 359 926 930 2018 29348365 10.1126/science.aar3247 PMC6080308

[36] Leary RJ Sausen M Kinde I et al Detection of chromosomal alterations in the circulation of cancer patients with whole-genome sequencing Sci Transl Med 4 162ra154 2012 10.1126/scitranslmed.3004742 PMC3641759 23197571

[37] Webb S: The cancer bloodhounds. Nat Biotechnol 34:1090-1094, 2016 [Erratum: Nat Biotechnol 35:178, 2017] 10.1038/nbt.3717 27824838

[38] Wyatt AW Annala M Aggarwal R et al Concordance of circulating tumor DNA and matched metastatic tissue biopsy in prostate cancer J Natl Cancer Inst 110 78 86 2018 10.1093/jnci/djx118 PMC6440274 29206995

[39] Beaver JA Jelovac D Balukrishna S et al Detection of cancer DNA in plasma of patients with early-stage breast cancer Clin Cancer Res 20 2643 2650 2014 24504125 10.1158/1078-0432.CCR-13-2933 PMC4024333

[40] Schweizer MT Antonarakis ES Liquid biopsy: Clues on prostate cancer drug resistance Sci Transl Med 7 312fs45 2015 10.1126/scitranslmed.aad4008 26537254

[41] Watson PA Arora VK Sawyers CL Emerging mechanisms of resistance to androgen receptor inhibitors in prostate cancer Nat Rev Cancer 15 701 711 2015 26563462 10.1038/nrc4016 PMC4771416

[42] Beltran H Prandi D Mosquera JM et al Divergent clonal evolution of castration-resistant neuroendocrine prostate cancer Nat Med 22 298 305 2016 26855148 10.1038/nm.4045 PMC4777652

[43] Ku SY Rosario S Wang Y et al Rb1 and Trp53 cooperate to suppress prostate cancer lineage plasticity, metastasis, and antiandrogen resistance Science 355 78 83 2017 28059767 10.1126/science.aah4199 PMC5367887

[44] Mu P Zhang Z Benelli M et al SOX2 promotes lineage plasticity and antiandrogen resistance in TP53- and RB1-deficient prostate cancer Science 355 84 88 2017 28059768 10.1126/science.aah4307 PMC5247742

[45] Jamaspishvili T Berman DM Ross AE et al Clinical implications of PTEN loss in prostate cancer Nat Rev Urol 15 222 234 2018 29460925 10.1038/nrurol.2018.9 PMC7472658

[46] de Bono JS De Giorgi U. Massard C et al PTEN loss as a predictive biomarker for the Akt inhibitor ipatasertib combined with abiraterone acetate in patients with metastatic castration-resistant prostate cancer (mCRPC) Ann Oncol 27 7180 2016 suppl 6

[47] Mateo J Carreira S Sandhu S et al DNA-repair defects and olaparib in metastatic prostate cancer N Engl J Med 373 1697 1708 2015 26510020 10.1056/NEJMoa1506859 PMC5228595

[48] Cheng HH Pritchard CC Boyd T et al Biallelic inactivation of BRCA2 in platinum-sensitive metastatic castration-resistant prostate cancer Eur Urol 69 992 995 2016 26724258 10.1016/j.eururo.2015.11.022 PMC4909531

[49] Brahmer JR Drake CG Wollner I et al Phase I study of single-agent anti-programmed death-1 (MDX-1106) in refractory solid tumors: Safety, clinical activity, pharmacodynamics, and immunologic correlates J Clin Oncol 28 3167 3175 2010 20516446 10.1200/JCO.2009.26.7609 PMC4834717

[50] Kwon ED Drake CG Scher HI et al Ipilimumab versus placebo after radiotherapy in patients with metastatic castration-resistant prostate cancer that had progressed after docetaxel chemotherapy (CA184-043): A multicentre, randomised, double-blind, phase 3 trial Lancet Oncol 15 700 712 2014 24831977 10.1016/S1470-2045(14)70189-5 PMC4418935

[51] Le DT Uram JN Wang H et al PD-1 blockade in tumors with mismatch-repair deficiency N Engl J Med 372 2509 2520 2015 26028255 10.1056/NEJMoa1500596 PMC4481136

[52] Guedes LB Antonarakis ES Schweizer MT et al MSH2 loss in primary prostate cancer Clin Cancer Res 23 6863 6874 2017 28790115 10.1158/1078-0432.CCR-17-0955 PMC5690834

[53] Pritchard CC Morrissey C Kumar A et al Complex MSH2 and MSH6 mutations in hypermutated microsatellite unstable advanced prostate cancer Nat Commun 5 4988 2014 25255306 10.1038/ncomms5988 PMC4176888

[54] Loriot Y Bianchini D Ileana E et al Antitumour activity of abiraterone acetate against metastatic castration-resistant prostate cancer progressing after docetaxel and enzalutamide (MDV3100) Ann Oncol 24 1807 1812 2013 23576708 10.1093/annonc/mdt136

[55] Noonan KL North S Bitting RL et al Clinical activity of abiraterone acetate in patients with metastatic castration-resistant prostate cancer progressing after enzalutamide Ann Oncol 24 1802 1807 2013 23585511 10.1093/annonc/mdt138

[56] Azad AA Eigl BJ Murray RN et al Efficacy of enzalutamide following abiraterone acetate in chemotherapy-naive metastatic castration-resistant prostate cancer patients Eur Urol 67 23 29 2015 25018038 10.1016/j.eururo.2014.06.045

[57] Brasso K Thomsen FB Schrader AJ et al Enzalutamide antitumour activity against metastatic castration-resistant prostate cancer previously treated with docetaxel and abiraterone: A multicentre analysis Eur Urol 68 317 324 2015 25108579 10.1016/j.eururo.2014.07.028

[58] Maughan BL Luber B Nadal R et al Comparing sequencing of abiraterone and enzalutamide in men with metastatic castration-resistant prostate cancer: A retrospective study Prostate 77 33 40 2017 27527643 10.1002/pros.23246

[59] Emamekhoo H Barata PC Edwin NC et al Evaluation of response to enzalutamide consecutively after abiraterone acetate/prednisone failure in patients with metastatic castration-resistant prostate cancer Clin Genitourin Cancer 16 429 436 2018 30236961 10.1016/j.clgc.2018.08.002

[60] Hu Y Ulrich BC Supplee J et al False positive plasma genotyping due to clonal hematopoiesis Clin Cancer Res 24 4437 4443 2018 29567812 10.1158/1078-0432.CCR-18-0143

